# Postoperative myocardial infarction after diagnostic video-assisted thoracoscopy and pleurodesis for catamenial pneumothorax: A unique case report

**DOI:** 10.4103/0019-5049.68388

**Published:** 2010

**Authors:** G Madhavi, N Satyanarayana

**Affiliations:** Department of Anaesthesiology, Government General and Chest Hospital, Hyderabad, Andhra Pradesh, India; 1 Department of Anaesthesiology, Osmania Medical College, Hyderabad, Andhra Pradesh, India

**Keywords:** Catamenial hemopneumothorax, intercostal drainage tube, myocardial infarction, perioperative myocardial infarction, recurrent pneumothorax, video-assisted thoracoscopy

## Abstract

Myocardial infarction (MI) is uncommon in patients undergoing noncardiac surgery without a history of coronary artery disease. But, patients with compromised pulmonary function and coexisting anaemia superimposed by precipitating factors like prolonged hypotension and tachycardia can culminate in myocardial catastrophe even in the absence of risk factors. We are herewith reporting an unusual case of postoperative non-ST elevation MI without any pre-existing ischemic heart disease. A 39-year-old female patient who was submitted for diagnostic video-assisted thoracoscopy and chemical pleurodesis for recurrent pneumothorax developed postoperative MI. After review of all the factors, it was found that the patient developed Type 2 MI as a sequel to oxygen supply and demand mismatch secondary to hypoxia and prolonged hypotension. This was evident in the 12-lead electrocardiogram and was confirmed by elevated cardiac biomarkers and regional wall motion abnormality on echocardiography.

## INTRODUCTION

In patients undergoing noncardiac surgery, the incidence of postoperative myocardial infarction (MI) without history suggestive of coronary artery disease is 0.6% of all surgical cases.[1] It was found that the incidence was 1.4% in veterans undergoing elective noncardiac surgery.[2] Most of the postoperative acute coronary events in such cases are secondary to imbalance created between oxygen supply and demand. It might be due to pre-operative factors like anaemia, hypertension and insulin-dependent diabetes or intra-operative factors like repeated hypertensive episodes, prolonged hypotension, tachycardia secondary to any reason and hypoxemia due to blood loss.

## CASE REPORT

A 39-year-old female patient was referred for evaluation of recurrent pneumothorax. She had suffered from attacks of pneumothorax intermittently over a period of 5 years. At every attack, she was admitted and treated with an intercostal drainage tube (ICDT) for 5–6 days. The patient was referred to our institute for definitive diagnosis and therapeutic intervention. In view of the recurrent nature of the symptoms with absence of any corroborative evidence of secondary pneumothorax, the patient was admitted for further investigation. The patient developed bloody discharge through the intercostal tube 2 days after admission, which was incidentally associated with menstruation. This prompted the diagnosis of catamenial hemopneumothorax, one of the manifestations of thoracic endometriosis. The patient’s chest X-ray showed a normal-sized heart, with the pneumothorax on the right side. A computerized tomographic scan revealed small bullous lesions at the apical region of the right lung in addition to pneumothorax. On fibreoptic bronchoscopy, brown-coloured mucosal pigmentation was seen in the right lower lobe bronchus, and biopsy from the lesion was inconclusive. It was decided to confirm the diagnosis by biopsy of the suspected lesions. The patient was posted for diagnostic video-assisted thoracoscopy (VAT) and chemical pleurodesis.

In her pre-anaesthetic assessment, the patient was found to be moderately built but anaemic, with a pulse rate (PR) of 82/mt, BP - 130/80 mmHg, respiratory rate - 18/mt, oxygen saturation (SpO_2_) - 97% on room air. Airway was adequate. The respiratory system examination showed decreased breath sounds on the right side with an ICDT *in situ*. Cardiovascular and other systemic examinations were unremarkable.

Her haemoglobin was 9 g and other biochemical parameters were normal. Resting electrocardiogram (ECG) was within normal limits. Echocardiography revealed a normal study.

The patient was scheduled for diagnostic VAT under general anaesthesia and lung isolation was contemplated as there was a possibility of difficulty in obtaining biopsy.

After shifting the patient into the theatre, a pulse oximeter, ECG and noninvasive blood pressure monitor were attached and basal readings showed a PR of 94/mt, SpO_2_ - 98%, BP - 134/82 mmHg and ECG tracing was normal. After securing venous line, Glycopyrrolate 0.2 mg and Fentanyl 100 μg were administered as premedication. Following pre-oxygenation, the patient was induced with thiopentone sodium at 5 mg/kg body weight and intubation with a left 35FG double-lumen tube was facilitated by succinyl choline 1.5 mg/kg. Isolation of lung was checked. Anaesthesia was maintained with intermittent positive-pressure ventilation supplemented with atracurium and sevoflurane. Thoracoscopy was performed and biopsy was taken from multiple sites. A small air leak was identified in the parenchyma of the apical region. After the biopsy, tetracycline slurry was instilled through intercostal drainage (ICD) for pleurodesis and ICD was clamped for facilitating its action. At this point, the SpO_2_ had dropped to 82–84%. The patient was immediately ventilated with 100% O_2_. It was suspected that the air leak from the apical segment might have increased the size of the pneumothorax, causing desaturation. Therefore, the clamp was removed, whereby the saturation improved to 95%. But, the extubation was delayed for 45 min in view of inadequate breathing attempts and as the blood pressure was on the lower side. The patient was shifted to the Post Anaesthesia Care Unit (PACU), with SpO_2_ 94%, BP - 100/60 mmHg and PR - 130/min.

In the PACU, the patient developed hypotension (70/50 mmHg) and the heart rate increased to 145–148/mt. Hypotension was initially corrected by volume repletion, which did not maintain haemodynamic stability. With the assumption that anaphylactic reaction could have occurred due to chemical pleurodesis, the patient was treated with hydrocortisone, subcutaneous adrenaline and chlorpheniramine. Later, noradrenaline drip was started in view of persistent tachycardia and resistant hypotension with which the blood pressure marginally improved to 100/60 mmHg. SpO_2_ was maintained at 96–97%, with oxygen supplementation at 5 L/min.

On the first postoperative day, the patient was anxious and complained of gradually increasing breathlessness, palpitation and profuse sweating. The patient’s SpO_2_ dropped to 92% in spite of oxygenation. A 12-lead ECG was taken and this showed sinus tachycardia with evidence of myocardial ischemia in the form of ST-depressions and T-wave inversions in leads I-III, aVF and V3-V6. Cardiac biomarkers showed elevated levels of Troponin T (0.12 ng/ml) and Creatine Kinase (CKMB – 94 IU/ml). Hence, the patient was shifted to the Intensive Critical Care Unit (ICCU) for further management.

The patient was diagnosed as acute coronary syndrome [Type1 perioperative MI (PMI)]. Her 2D-ECHO revealed regional wall motion abnormality of left ventricle, hypokinesis of the entire septum and moderate LV dysfunction, with an ejection fraction of 41%. She was stabilized with beta blockers, digitalis, anticoagulants, antiplatelet agents, nitrates and oxygen supplementation. She was transfused with packed RBCs after stabilizing for 48 h and was discharged with stable cardiovascular status with an advice of coronary angiogram at a later date.

## DISCUSSION

PMI is a major cause of morbidity and mortality in patients undergoing noncardiac surgery. MI secondary to ischemia due to either increased oxygen demand or decreased supply, e.g. coronary spasm, coronary embolism, anaemia, arrhythmias, hypertension or hypotension, is classified as type 2 MI.[3,4]

Risks for postoperative MI are poor pre-operative cardiac status, postoperative hypotension and new intra-operative ST-T changes.[5] Such factors should direct us to make a plausible diagnosis by excluding common causes and, ultimately, suspect the possibility of PMI.

Unlike previous studies, the recent evidence suggests that PMI occurred earlier, with most of the events occurring on the day of surgery or the first postoperative day, and is most often asymptomatic, non-Q wave and ST-segment depression type.[6] Troponin levels of at least one value above the 99th percentile is diagnostic of MI.[7]

Our patient developed an acute coronary event perioperatively (Type 2 PMI) in the absence of risk factors, which is attributed to mismatch in oxygen supply and demand.[8] The mismatch was secondary to (1) pre-existing lung pathology, (2) single lung anaesthesia and (3) increasing pneumothorax following intra-operative ICD clamping, leading to hypoxia. It can also be postulated that anaphylaxis due to chemical pleurodesis, persistent postoperative hypotension and sinus tachycardia might have added fuel to the fire. Coexisting anaemia due to recurrent episodes of menorrhagic cycles have contributed to mismatch in oxygen supply and demand, ultimately culminating in myocardial catastrophe. Thus, we conclude that multiple factors might have precipitated the myocardial ischemic event in this case.

There was a delay in the diagnosis because PMI was silent and breathlessness was attributed to coexisting pulmonary pathology. But, the 12-lead ECG changes in concurrence with elevated Troponin T and CKMB levels alerted us to shift the case to the ICCU and, ultimately, the patient recovered due to timely intervention.

This clearly suggests that whenever there is an oxygen supply and demand mismatch due to various factors as already discussed, MI can occur, and a vigilant anaesthesiologist should be aware of these consequences. Even with diagnostic thoracoscopic procedures such complications are likely. It is also crucial that pre-existing anaemia should be corrected before contemplating thoracic surgery, especially with pre-existing lung pathology, where the oxygen-carrying capacity can be compromised.
